# Improving Access to HLA-Matched Kidney Transplants for African American Patients

**DOI:** 10.3389/fimmu.2022.832488

**Published:** 2022-03-24

**Authors:** Dulat Bekbolsynov, Beata Mierzejewska, Sadik Khuder, Obinna Ekwenna, Michael Rees, Robert C. Green, Stanislaw M. Stepkowski

**Affiliations:** ^1^Department of Medical Microbiology and Immunology, University of Toledo, Toledo, OH, United States; ^2^Department of Medicine and Public Health, University of Toledo, Toledo, OH, United States; ^3^Department of Urology, College of Medicine, University of Toledo, Toledo, OH, United States; ^4^The of Alliance for Paired Donation, Maumee, OH, United States; ^5^Department of Computer Science, Bowling Green State University, Bowling Green, OH, United States

**Keywords:** kidney transplantation, transplant survival, race, allocation, human leukocyte antigen, human leukocyte antigen mismatch, immunogenicity

## Abstract

**Introduction:**

Kidney transplants fail more often in Black than in non-Black (White, non-Black Hispanic, and Asian) recipients. We used the estimated physicochemical immunogenicity for polymorphic amino acids of donor/recipient HLAs to select weakly immunogenic kidney transplants for Black vs. White or non-Black patients.

**Methods:**

OPTN data for 65,040 donor/recipient pairs over a 20-year period were used to calculate the individual physicochemical immunogenicity by hydrophobic, electrostatic and amino acid mismatch scores (HMS, EMS, AMS) and graft-survival outcomes for Black vs. White or vs. non-Black recipients, using Kaplan-Meier survival and Cox regression analyses. Simulations for re-matching recipients with donors were based on race-adjusted HMS thresholds with clinically achievable allocations.

**Results:**

The retrospective median kidney graft survival was 12.0 years in Black vs. 18.6 years in White (6.6-year difference; p>0.001) and 18.4 years in non-Black (6.4-year difference; p>0.01) recipients. Only 0.7% of Blacks received transplants matched at HLA-A/B/DR/DQ (HMS=0) vs. 8.1% in Whites (p<0.001). Among fully matched Blacks (HMS=0), graft survival was 16.1-years and in well-matched Blacks (HMS ≤ 3.0) it was 14.0-years. Whites had 21.6-years survival at HMS ≤ 3.0 and 18.7-years at HMS ≤ 7.0 whereas non-Blacks had 22.0-year at HMS ≤ 3.0 and 18.7-year at HMS ≤ 7.0, confirming that higher HMS thresholds produced excellent survival. Simulation of ABO-compatible donor-recipient pairs using race-adjusted HMS thresholds identified weakly immunogenic matches at HMS=0 for 6.1% Blacks and 18.0% at HMS ≤ 3.0. Despite prioritizing Black patients, non-Black patients could be matched at the same level as in current allocation (47.0% vs 56.5%, at HMS ≤ 7.0).

**Conclusions:**

Race-adjusted HMS (EMS, AMS)-based allocation increased the number of weakly immunogenic donors for Black patients, while still providing excellent options for non-Black recipients.

## Introduction

African American (Black) patients are disadvantaged in the outcome of their kidney transplants not only when compared to Caucasian (White) but also to Hispanic or Asian (non-Black) patients (1). According to the United Network of Organ Sharing (UNOS), Black patients constitute 32% of all patients with end-stage renal disease (ESRD) and 31% of patients on the UNOS waiting list, while only 12.4% of the total U.S. population (2). Black recipients also suffer from a higher frequency of transplant-related complications, such as delayed graft function, acute graft failure, and higher risk of graft loss (3, 4). These differences have been attributed to socioeconomic factors, and genetic factors such disease-predisposing polymorphisms in non-muscle myosin heavy chain 9 (MYH9) and apoliprotein L1 (ApoL1) genes (5, 6), and to reduce *de-novo* donor-specific antibody production (7).

Donor/recipient matching at Human Leukocyte Antigen (HLA) has been shown to improve graft survival (8, 9). Similar benefits were observed in Black recipients, despite the fact that the HLA matching is harder for Black than White patients (10). Though OPTN policy mandates sharing of fully HLA-A/B/DR-matched kidneys (zero mismatch, 0-MM), Black patients are less likely to receive a 0-MM donor, and therefore the vast majority of 0-MM kidneys are transplanted into patients of other races (11). Only rarely do inter-racial transplants result in 0-MM bone marrow transplants (12). Consequently, HLA-based allocation systems disadvantage Black patients.

Estimations of HLA diversity among races demonstrated the low likelihood of providing HLA-matched transplants for both Blacks and patients of other races (10). However, our recent publication showed that the hydrophobic mismatch score (HMS), based on a continuous HLA amino acid donor/recipient disparity scale, provided a unique opportunity to adjust HMS thresholds (13). Therefore, we propose replacing the 6-integer A-B-DR analog HLA-based matching (14, 15) with a linear HMS scale utilizing race-adjusted thresholds. The HMS scale was used as it produced similar results as electrostatic and amino acid mismatch scores calculated by the previously published Cambridge algorithm (14, 15). Our hypothesis is that a race-adjusted HMS threshold significantly improves access to weakly immunogenic transplants for Black patients without negatively impacting White, Hispanic, and Asian patients. Our results confirmed that Black recipients were transplanted over the last 20 years with significantly higher immunogenic donors than White, Hispanic, and Asian recipients. The HMS-based scale allowed for re-matching of Black patients with to immunogenic donors in a simulated matching algorithm without worsening choices for other races. Our simulations suggest that an alternative allocation system using race-adjusted HMS thresholds could improve access to weakly immunogenic transplants for Black patients, while simultaneously lowering mean HMS for White, Hispanic, and Asian patients.

## Materials and Methods

### Patient Population and HLA Immunogenicity Quantitation

Out of 311,558 deceased donor transplants in the Scientific Registry of Transplant Recipients (SRTR) (16), we derived 114,420 records with complete HLA-A/B/DR split antigens record, race information and physicochemical HLA immunogenicity imputable using the Cambridge algorithm. We then narrowed it to 65,040 adult first-time kidney transplants in 1/1/2000-12/1/2019. Serological HLA types were converted from to high resolution using the HaploStats algorithm (17). Imputation accuracy was verified using the clinically typed patient cohort (17); HLA immunogenicity was quantified for each donor/recipient pair by the Oxford algorithm to obtain hydrophobic (HMS), electrostatic and amino acid mismatch score (14, 15).

This study used data from the Scientific Registry of Transplant Recipients (SRTR). The SRTR data system includes data on all donor, wait-listed candidates, and transplant recipients in the US, submitted by the members of the Organ Procurement and Transplantation Network (OPTN). The Health Resources and Services Administration (HRSA), U.S. Department of Health and Human Services provides oversight to the activities of the OPTN and SRTR contractors. The principles of the Helsinki Declaration were followed for steps involving patient information.

All analyses were performed in SAS 9.4 (SAS Institute Inc., Cary, NC) and in R (R Foundation for Statistical Computing, Vienna, Austria).

### Proportional Hazard Ratio Model

The impact of immunogenicity in the context of potential clinical confounders was done by semi-parametric Cox proportional hazard regression models (Cox PHR). Confounder variables were selected by building univariate regression models with initial sets of confounder variables, and variables showing significant association with graft survival in multiple steps of purposeful selection were included in the multiple Cox regression model (18).

The selected variables were tested for the assumption of proportional hazard distribution by simulating possible score process components versus follow-up time plots for each confounder (19). The proportional hazard distribution was also inferred based on these plots using the Kolmogorov-type supremum test. Variables violating the proportionality assumption were included with time log transformation.

### Survival Analyses

Non-parametric Kaplan-Meier survival probability estimates were performed to compare graft failure rates. Comparison groups were matched to account for the possible differences in clinical confounder distribution (20). Pairwise matching using confounders identified in Cox PHR models was done in R using the MatchIT package (21).

### Simulated Allocation Model

A simulation model was developed to provide preliminary confirmation that the methods proposed could have the expected impact. The Python-based simulation takes the complete list of donor-recipient pairs and separates them into two individual lists of donors and recipients. Recipients used in re-matching are randomly selected from the entire pool of recipients based on the required proportions of each race included in a given simulation, while donors are randomly selected with no consideration given to race. The distribution of recipient race in the pool was reflective of the race distribution in patients on the UNOS transplant waiting list: 36% Caucasian, 32% African American, 22% Hispanic, 10% Asian. Recipients are then matched to the best donor available using an iterative search beginning with the first recipient. Goodness of match is determined using ABO and either a specified HMS scale up to a given threshold or mismatch score. When considering HMS, the simulation iteratively finds best matches for those with a HMS score less than 0, then 1, then 2, and so on until the threshold is reached. As this simulation is a proof of concept, date of the original transplant was not considered.

## Results

### Study Design and Oversight

The study was designed by the first and the last two authors. All participated investigators reviewed the data, which were analyzed by the first three authors and the last two authors. The first and last authors wrote the manuscript and vouch for the accuracy and completeness of the data. All authors supported the submission and guarantee the fidelity of the study with the protocol. The NIH sponsored the study did not place confidentiality restrictions on authors or institutions involved in this study.

### The Accuracy of Imputation of High-Resolution HLA Types

The HaploStats algorithm was used to impute high-resolution HLA-A/B/DRB1/DQB1 types (17, 22) based on the frequency distribution in the race (23, 24). The accuracy of imputation was confirmed on 1,095 Caucasian transplant candidates at Queen Elizabeth II Health Sciences Centre, Halifax, Canada typed at HLA-A/B/DRB1/DQB1 loci using a combination of sequence based typing and extended region SSO methods (13); The 1,095 HLA genotypes were used to form 547 simulated donor/recipient pairs. Their serological HLA split antigen data was used to impute the likely high-resolution HLA types, and the two sets of HLA types were compared. The imputed 4-digit types were 96% identical for HLA-A, 92% for -B, 73% for -DRB1, and 85% for -DQB1 with the high-resolution types received in laboratory. Furthermore, when the HMS, EMS, AMS were calculated for these 547 donors/recipient pairs using their real high-resolution HLA-types, were compared to the scores calculated using their imputed high-resolution HLA types in paired t-test, no difference was found (p=0.31), see ref (13).. In addition, comparison of trimmed HMS, EMS and AMS values calculated based on either real or imputed high-resolution types all produced R^2^-values of 0.99 showing little, if any, impact of HLA genotype imputation on the proof-of-concept results (**Supplemental Figure 1**).

### Study Population and Definitions

The cohort included 65,040 first-time deceased donor transplants: 22,781 Black (35.0%), 27,550 White (42.3%), 9,923 Hispanic (15.3%), and 3,945 Asian (6.1%) recipients, and 841 recipients of other races (1.3%). Black patients more often spent over 5 years on dialysis (39.0% vs 22.8%; p<0.001), were younger (30.9% under 34 years vs 22.6%; p<0.001), more sensitized (2.0% at PRA≥95% vs 1.5%, p<0.001) and less frequently had private insurance (p>0.001) compared to non-Black recipients (**Table 1**).

**Table 1:** Demographic and clinical characteristics of patients included in the analysis.

Variable	Blacks	non-Blacks	p-value*
Time on dialysis			<0.0001
Less than 1 year	627 (2.8%)	2,815 (6.7%)	
1 to 3 years	3,902 (17.1%)	9,404 (22.3%)	
3 to 5 years	5,337 (23.4%)	8,722 (20.6%)	
Over 5 years	8,894 (39.0%)	9,648 (22.8%)	
Unknown or not applicable	4,021 (17.7%)	11,670 (27.6%)	
Recipient age at transplant			<0.0001
18-34 years	7,032 (30.9%)	9,532 (22.6%)	
35-49 years	2,483 (10.9%)	4,027 (9.5%)	
50-64 years	9,707 (42.6%)	18,296 (43.3%)	
65 years and higher	3,559 (15.6%)	10,404 (24.6%)	
Peak PRA level			<0.0001
0-19%	9,486 (41.6%)	19,134 (45.3%)	
20-79%	2,648 (11.6%)	3,784 (8.9%)	
80-94%	596 (2.6%)	969 (2.3%)	
95-100%	438 (2.0%)	639 (1.5%)	
Unknown	9,613 (42.2%)	17,733 (42.0)	
Pre-transplant dialysis			<0.0001
No dialysis	2,362 (10.4%)	8,056 (19.1%)	
Hemodialysis	11,472 (50.4%)	17,240 (40.8%)	
Peritoneal dialysis	1,617 (7.1%)	3,924 (9.3%)	
Other or unknown	7,330 (32.1%)	13,039 (30.8%)	
Recipient race			<0.0001
White	0 (0%)	27,550 (65.2%)	
Black	22,781 (100%)	0 (0%)	
Hispanic/Latino	0 (0%)	9,923 (23.5%)	
Asian	0 (0%)	3,945 (9.3%)	
Other	0 (0%)	841 (2.0%)	
Donor race			<0.0001
White	16,834 (73.9%)	34,985 (82.8%)	
Black	5,268 (23.1%)	5,451 (12.9%)	
Hispanic/Latino	74 (0.3%)	256 (0.4%)	
Asian	497 (2.2%)	1,232 (2.9%)	
Other	74 (0.5%)	435 (1.0%)	
Candidate BMI > 30			<0.0001
Yes	8,803 (38.6%)	13,307 (31.5%)	
No	13,679 (60.1%)	28,499 (67.4%)	
Unknown	299 (1.3%)	453 (1.1%)	
Donor BMI > 30			0.0079
Yes	6,761 (30.7%)	12,123 (28.7%)	
No	16,020 (70.3%)	30,136 (71.3%)	
Donor gender			0.1308
Male	13,531 (59.4%)	24,842 (58.8%)	
Female	9,250 (40.6%)	17,417 (41.2%)	
Recipient gender			0.0007
Male	13,733 (60.3%)	26,052 (61.6%)	
Female	9,048 (39.7%)	16,207 (38.4%)	
Recipient’s primary source of payment			<0.0001
Private insurance	7,582 (33.3%)	18,706 (44.3%)	
Other	15,156 (66.5%)	23,519 (55.6%)	
Unknown	43 (0.2%)	34 (0.1%)	
Cold ischemia time > 24 hours			0.4356
Yes	8,469 (37.2%)	15,563 (36.8%)	
No	13,270 (58.3%)	24,716 (58.5%)	
Unknown	1,042 (4.5%)	1,980 (4.7%)	
Maintenance immunosuppression			0.0023
CsA/Aza/Pred	4,381 (19.2%)	8,147 (19.3%)	
CsA/MMF/Pred	1,444 (6.3%)	2,552 (6.0%)	
CsA/Pred	786 (3.5%)	1,375 (3.3%)	
TAC/MMF/Pred	8,797 (38.6%)	15,972 (37.8%)	
TAC/MMF	292 (1.3%)	489 (1.2%)	
TAC/Pred	211 (0.9%)	400 (0.9%)	
MMF/Pred	242 (1.1%)	472 (1.1%)	
Other	989 (4.3%)	1,744 (4.1%)	
No data	5,639 (24.8%)	11,108 (26.3%)	
HLA-A/B/DR mismatch			<0.0001
0	347 (1.5%)	3,360 (8.0%)	
1	79 (0.4%)	430 (1.0%)	
2	517 (2.2%)	2,021 (4.8%)	
3	2,228 (9.8%)	5,638 (13.3%)	
4	5,901 (25.9%)	10,864 (25.7%)	
5	8,744 (38.4%)	13,086 (31.0%)	
6	4,876 (21.4%)	6,347 (15.0%)	
Unknown	89 (0.4%)	513 (1.2%)	

*Chi-squared test.

### Higher Donor HLA Immunogenicity in Black Than in White Recipients

**Figure 1:**
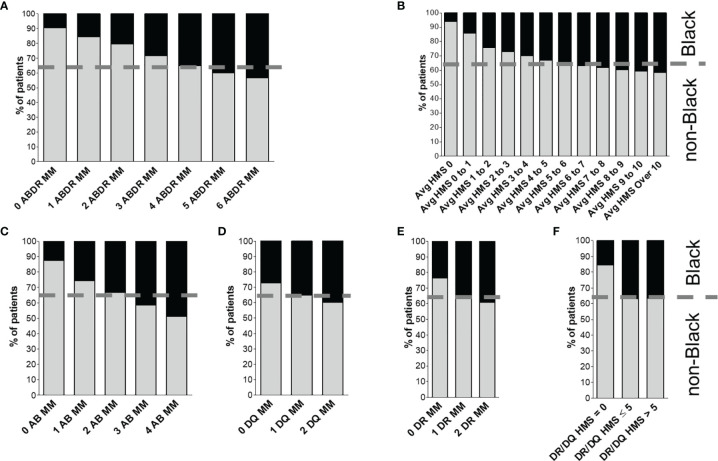
Distribution of donor/recipient immunogenicity among Black and non-Black recipients. Patients were stratified either by HLA-A/B/DR mismatch **(A)** or by average HLA-A/B/DR/DQ HMS level **(B)**. Class I immunogenicity as HLA-A/B antigenic mismatch distribution is shown in **(C)**, and class II is shown as distribution of mismatches in HLA-DQ **(D)** and -DR **(E)** panels. Finally, panel **(F)** shows distribution of HLA-DR/DQ HMS scores. The dotted line represents the proportion of non-Black and Black recipients in the cohort.

Disproportionately fewer Black recipients were transplanted with 0-, 1-, 2- or 3-HLA MM kidney transplants than White and non-Black recipients (**Figure 1A**). Black recipients constituted 35.0% of the cohort but only 1.5% of them received fully matched at HLA-A/B/DR (MM=0) transplants (**Table 1**). Non-Black recipients constituted 65.0% of the cohort, and about 8.0% of them received 0-MM transplants (**Table 1**). In contrast, more highly immunogenic transplants (with 4-, 5- and 6-HLA MM) were transplanted to Black (85.7%) compared to non-Black (71.7%) recipients (**Figure 1A**).

A similar pattern was seen with the HMS scale (**Figure 1B**). Fewer weakly immunogenic (HMS=0, 0.7% vs 5.9%) and more highly immunogenic (HMS>7, 53.7% vs 43.5%) kidneys were transplanted into Black recipients (**Figure 1B**); breakdown by HLA class (HLA-A/B, HLA-DR and -DQ) produced similar results (**Figures 1C–F**). Black recipients were disadvantaged in every analysis.

### Impact of HLA Immunogenicity on Graft Survival in Black vs. White and Non-Black Recipients

**Figure 2:**
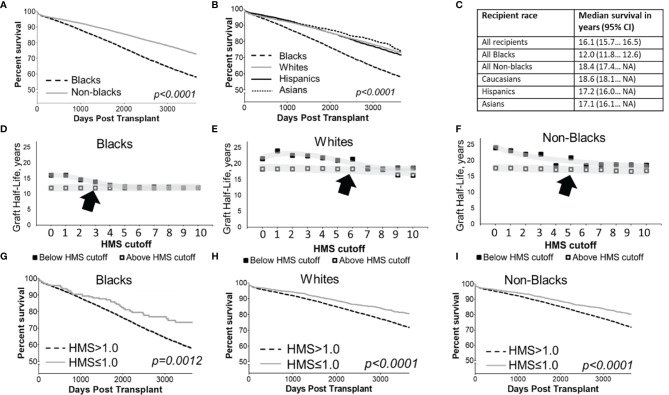
Kaplan-Meier death-censored survival estimates for deceased donor kidney allograft in cohort patients stratified by recipient race **(A, B)** and summary of their median survival with 95% confidence intervals **(C)**. Median death-censored graft survival (graft half-lives) in Blacks **(D)**, Whites **(E)** and non-Blacks **(F)** measured in various immunogenicity categories defined HLA-A/B/DR/DQ HMS less than or equal to integer cutoff values from 0 to 10 are shown. Each immunogenicity category includes all patients with HMS scores below its threshold, which means each category overlaps with categories under lower HMS thresholds. Death-censored graft survival in Black patients **(G)**, as well as White **(H)** and all non-Black patients **(I)** stratified by antigenic HLA mismatch is shown. Arrows show acceptable HMS values associated with the good transplant survival.

Overall, the graft survival half-life in Black recipients was 12.0 years vs. 18.6 years in White recipients (**Figures 2A–C**), an over 6-year gap, 17.2 for Hispanic, a 5.2-year difference (p>0.01), and 17.1 years for Asian, a 5.1-year difference (p>0.001; **Figures 2B, C**). Stratification by integer 0-10 thresholds of HMS, EMS or AMS scores confirmed the unequal survival profile (**Figure 2**; **Supplemental Table 1**); Low HLA immunogenicity was associated with better transplantation outcomes: Black recipients reached a 16.1-year graft survival at HMS=0 and HMS ≤ 1.0; 14.6 years at HMS ≤ 2.0, and 14.0 years at HMS ≤ 3.0, higher HMS thresholds had substantially worse survivals (**Supplemental Table 1**). For White recipients, graft survival as a function of immunogenicity was better: 21.6 years was at HMS=0; and all remaining HMS values up to 10.0 were 15.0 years or better (**Supplemental Table 1**). Similar pattern of 15-year survival were observed for all non-Black patients with HMS ≤ 10.0.

Since the best projected survival in Black recipients was 16.1 years (at HMS, EMS or AMS ≤ 1.0), compared to the current median survival of 12.0 years and 13.9 years at HMS ≤ 3.0, we used the HMS ≤ 1.0 as an optimal and the HMS ≤ 3.0 an acceptable threshold for Black recipients (**Figure 2G**). Stratification based on EMS and AMS scores produced similar results that are not shown. Graft survival in well-matched White and all non-Black patients with HMS=0 was better than in the same patients with 0-/1-HLA MM (24.1 vs 22.9 years for Whites, 23.1 vs 22.1 years for non-Blacks). Black patients are hard to match at HMS=0 due to high diversity of their HLA cluster, therefore, HMS ≤ 3.0 was accepted as an immunogenicity threshold producing better transplantation outcomes for this race (13.9 years). Notably, three times as many Black recipients were in HMS ≤ 3.0 (n=1,252) than in 0-/1-HLA MM (n=347) cohort. The difference in thresholds for Black vs. non-Black cohort created the basis to build the equality model to select donors. For further analyses aimed at finding maximum numbers of well-matched donors, both thresholds of HMS=0 and HMS ≤ 3.0 were used.

Because racial cohorts differ in variables (**Table 1**), we used multiple Cox regression models to gauge the impact of HLA immunogenicity with variables on graft survival (**Supplemental Table 2**). Initially, variables were tested individually for each cohort with an assumption for proportional hazards distribution (**Supplemental Table 3**).

To show the difference in graft survival between Black and White patients in the context of other clinical confounders, we applied a statistical matching method for Kaplan-Meier survival analysis. For this survival analysis, samples of Black and White recipients were adjusted to be similar in the variables shown in **Supplemental Table 3**. After achieving pairwise matching the Kaplan-Meier showed 10.9-year graft survival in Black recipients vs. 13.5-year graft survival in White and 14.0-year non-Black recipients (**Supplemental Figure 2**).

### Impact of Immunogenicity in -DR/-DQ Loci on Graft Survival in Black and Non-Black Recipients

HLA-DR matching alone improved allograft survival in Blacks to 12.9 years (MM=0) vs. 11.8 years (MM=2, p=0.085) and in non-Blacks to 19.3 years (MM=0) vs. 16.6 years (MM=2, p<0.001);. When Black patients were matched at -DR/-DQ, the survival improved slightly to 12.8 years, though sample size was too small to achieve statistical significance (p=0.444, **Supplemental Figure 3A**). A similar trend was seen in non-Black recipients (p<0.001, **Supplemental Figure 3B**). The most optimal survival of 16.1 years (HMS ≤ 1.0) was produced for HLA-A/B/DR/DQ loci in Blacks vs. 12.0 years for HMS>1.0 (p<0.001; **Supplemental Table 1**). Similarly, for non-Blacks, the HMS ≤ 2.0 for HLA-A/B/DR/DQ achieved 24.0 years vs. 17.7 years with HMS>2.0 (p<0.001, **Supplemental Table 1**). These already excellent survival times were confirmed by a covariate matched population of 16.1 years at HMS=0 in Black and 17.4 years at unadjusted non-Black recipients (**Supplemental Figure 3C**).

### Improving Access to Weakly Immunogenic Transplants for Black Patients

**Figure 3:**
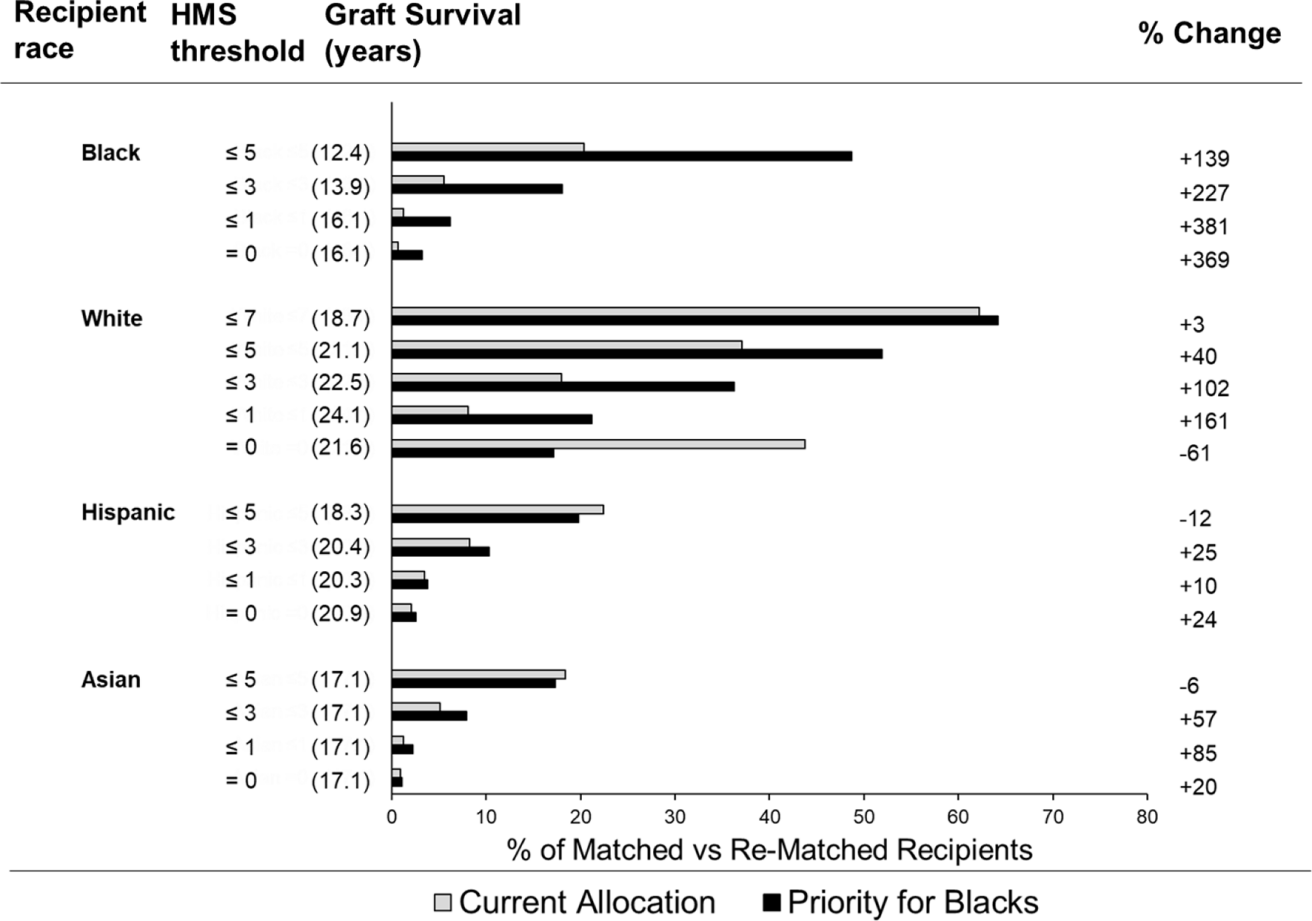
Percent of different ethnicity patients who receive a transplant under the indicated HMS threshold using current allocation vs. simulated allocation with priority for Black recipients. Bar graphs represent the percent of patients of various races who received a transplant under the indicated HMS threshold. The percent of patients who receive a transplant under current allocation policy (grey bars) vs. simulated allocation with the priority for Black patients (black bars).

In the current allocation system only 6% of deceased donor transplants are fully matched (25), while 83.6% of transplants have at least three HLA-A/B/DR mismatches. To improve matching, we used our continuous immunogenicity system to simulate kidney allocation among mixed 5,000 Black (32%), White (36%), Hispanic (22%) and Asian (10%) recipients, reflecting a race distribution of recipients in 2020. In each scenario, one race was prioritized to get the lowest HMS transplants, with the remaining races receiving donors from the remaining pool. When the randomly generated donor-recipient pair had immunogenicity higher than a designated threshold, it was re-matched until the weakly immunogenicity requirement was satisfied. ABO compatibility was enforced. When Blacks were prioritized, 3.2% Black, 17.2% White, and 9.7% non-Black recipients found transplants with HMS=0 (**Table 2**); their projected graft survivals were 16.1 years for Blacks, 21.6 years for Whites, and 21.50 years for non-Blacks (**Supplemental Table 1**). For comparison, currently only 0.7% Blacks, 8.1% Whites, and 5.9% non-Blacks are matched at this level. When the same priority was extended to HMS ≤ 3.0, 18.0% Blacks, 36.3.% Whites, and 23.2% non-Blacks were rematched. Today, only 5.5% Black, 18.0% Whites, and 14.3% non-Blacks are matched at HMS ≤ 3.0 with the current allocation policy. The median graft survival for Blacks at HMS ≤ 3.0 was 13.9 years (**Supplemental Table 1**). When HMS ≤ 7.0 was used just for non-Black patients, 64.2% Whites and 56.5% non-Black patients were matched with identical 18.7-year graft survivals for White and non-Black patients (**Supplemental Table 1**). Interestingly, the priority for both Whites or non-Blacks made little improvement for them but eliminated most weakly immunogenic transplants in Black recipients (**Figure 3**; **Table 2**).

**Table 2:** Percentage of Black and non-Black patients that currently receive or could receive under the tested allocation policy transplants with HMS below the indicated threshold.

HMS threshold	Retrospective median survival, years		Percent of patients receiving transplants with HMS below these thresholds					
			Current allocation policies		Blacks prioritized		Whites prioritized	
	Blacks	non-Blacks	Blacks	non-Blacks	Blacks	non-Blacks	Blacks	non-Blacks
HMS = 0	16.1	24.0	0.7	5.9	3.2	9.7	1.0	11.6
HMS ≤ 1.0	16.1	23.1	1.3	7.8	6.2	12.3	2.1	15.3
HMS ≤ 2.0	14.6	21.9	2.6	10.1	11.0	17.3	4.4	21.4
HMS ≤ 3.0	13.9	22.0	5.5	14.3	18.0	23.2	8.4	28.3
HMS ≤ 4.0	12.9	18.4	11.1	21.3	26.0	29.5	13.4	35.8
HMS ≤ 5.0	12.4	21.0	20.4	31.5	33.8	35.8	19.1	42.7
HMS ≤ 7.0	11.9	18.7	46.3	56.5	48.7	47.0	30.4	55.0

If the median graft survival in each immunogenicity category is multiplied by the number of recipients, the resulting kidney life-years (KLY) can be used as a measure of outcomes of kidney transplantation (26). We then multiplied the projected graft survival in years by the number of transplants with our race-specific HMS thresholds: Black recipients gained 2,300 KLY at HMS=0 over their current outcomes, 4,400 KLY at HMS ≤ 1.0, and 9,800 KLY at HMS ≤ 3.0. Even with prioritization of Blacks, Whites gained 13,400 KLY at HMS=0, and 28,000 KLY at HMS ≤ 3.0, with similar outcomes for Hispanics and Asians (**Supplemental Table 3**).

## Discussion

We demonstrate how the current allocation system produces a disparity of immunogenicity and graft survival for Black patients receiving deceased donor kidney transplants in the United States. We further provide simulation of a novel allocation approach that suggests improved allocation equity is achievable. Our analysis revealed four main findings: 1) the current kidney allocation system leads to Black recipients receiving kidney transplants that are more immunogenic than White or non-Black recipients; 2) the simulated allocation based on race-adjusted HMS thresholds benefited Black recipients without harming White and all non-Black recipients; 3) the best results were observed by HMS matching at HLA-A/B/DR/DQ loci with race-adjusted HMS thresholds; and, 4) HMS-based matching improve the survival of kidney allografts for Black recipients.

There are several reports confirming that Black recipients have significantly shorter kidney graft survivals and more frequently experience allograft rejection than White recipients (1, 3–6, 27). In one report, Black patients had 5-year graft survival of 69% vs. 83% in White patients (28). On one hand, this difference was correlated with non-immunological reasons such as an access to medical care and socioeconomic status of recipients (29). Interestingly, recent stratification by three income levels showed similar outcomes in three Black groups (30). On the other hand, Black race was also associated with several immunological factors affecting transplant survival, such as subtherapeutic tacrolimus levels (31), as well as genetic variants in cytochrome 450 (32), and apoliprotein L1 (ApoL1) genes (6). In addition, comorbidities such as diabetes and hypertension are co-dominantly associated with Black race and with graft failure (33). Most likely, all these factors contribute to worse clinical outcomes in the kidney allograft survival of Black recipients.

Due to the lack of *bona fide* data on high-resolution HLA types, we had to rely on available serological HLA types with different race composition to impute their most likely high-resolution types. This technique had been used in the number of studies before (34). However, in our case we were specifically interested in Black patients, whose HLA diversity is known to be higher compared to whites (35). Although we went to great effort to verify the accuracy of high-resolution HLA types imputation in our database (**Supplemental Figure 1**), we did not directly verified this accuracy for Blacks. An indirect way of comparing HLA type imputation accuracy in HaploStats may be found by comparing the likelihood values or typing ambiguity scores for each individual imputation that the algorithm provides (36). We believe that the accuracy of our imputation method is sufficient for the conclusions about the impact of race on kidney allograft survival. Furthermore, we plan to verify imputation for Black patients.

Increasing molecular disparities in hypervariable HLA region present an underlying mechanism for anti-donor humoral response and allograft rejection (37). In addition to HLA-A/B/DR disparities, the -DQ antigens, measured as immunogenic eplets, were responsible for the potent antibody response to kidney allografts (38, 39), as well as the incidence of graft-versus-host (HVG) disease after hematopoietic stem cell transplantation (40). The total number of immunogenic eplets as an eplet load is currently viewed as a clinically relevant measure of the immune response intensity, and therefore clinicians perform an “epitope matching” (41). The HMS values correlated with eplet load (9) and with the antibody response to kidney transplants (42).

The proposed HMS scale is a realistic method to adjust HLA immunogenicity in a race-adjusted fashion. The current allocation system inadvertently matches Black patients to donors with significantly higher immunogenic transplants compared to other races, this was not the case in our simulated allocation. However, prioritizing Blacks for HLA matching and using race-adjusted immunogenicity thresholds in allocation would result in a net gain of thousands additional kidney life-years for all races, as shown in **Supplemental Table 4**. Thus, this conceptual solution for a race-adjusted HMS-based matching of donors provided significant improvement for Black patients without impairing chances for other races.

## Data Availability Statement

Publicly available datasets were analyzed in this study. This data can be found here: The Scientific Registry of Transplant recipients [www.srtr.org].

## Author Contributions

DB, BM, MR, and SS guided the research and wrote the manuscript. RG performed high-resolution HLA types imputation and re-matching simulation analyses. SK consulted and performed the statistical analyses. OE provided intellectual support for data processing and analyses. All authors contributed to the article and approved the submitted version.

## Funding

This work was funded by the National Institutes of Health/National Library of Medicine grant #N-127429-01. Additional funding was provided by The Alliance for Paired Donation, Maumee, OH, USA.

## Author Disclaimer

The SRTR data include all donors, wait-list candidates, and transplant recipients submitted by the members of the Organ Procurement and Transplantation Network (OPTN). The Health Resources and Services Administration (HRSA) is the U.S. Department of Health and Human Services providing an oversight to the activities of the OPTN and SRTR contractors.

## Conflict of Interest

The authors declare that the research was conducted in the absence of any commercial or financial relationships that could be construed as a potential conflict of interest.

## Publisher’s Note

All claims expressed in this article are solely those of the authors and do not necessarily represent those of their affiliated organizations, or those of the publisher, the editors and the reviewers. Any product that may be evaluated in this article, or claim that may be made by its manufacturer, is not guaranteed or endorsed by the publisher.
