# Supplementary material for: Improving Access to HLA-Matched Kidney Transplants for African American Patients
Source: Front Immunol. 2022 Mar 24;13:832488. doi: 10.3389/fimmu.2022.832488 (PMC8989073; doi:10.3389/fimmu.2022.832488)
Supplement: Supplementary file 1 [file DataSheet_1.docx]

Supplementary Material

# Supplementary Data

Supplementary Material should be uploaded separately on submission. Please include any supplementary data, figures and/or tables. All supplementary files are deposited to FigShare for permanent storage and receive a DOI.

Supplementary material is not typeset so please ensure that all information is clearly presented, the appropriate caption is included in the file and not in the manuscript, and that the style conforms to the rest of the article. To avoid discrepancies between the published article and the supplementary material, please do not add the title, author list, affiliations or correspondence in the supplementary files.

# Supplementary Figures and Tables

For more information on Supplementary Material and for details on the different file types accepted, please see [here](http://home.frontiersin.org/about/author-guidelines#SupplementaryMaterial). Figures, tables, and images will be published under a Creative Commons CC-BY licence and permission must be obtained for use of copyrighted material from other sources (including re-published/adapted/modified/partial figures and images from the internet). It is the responsibility of the authors to acquire the licenses, to follow any citation instructions requested by third-party rights holders, and cover any supplementary charges.

## Supplemental Figures


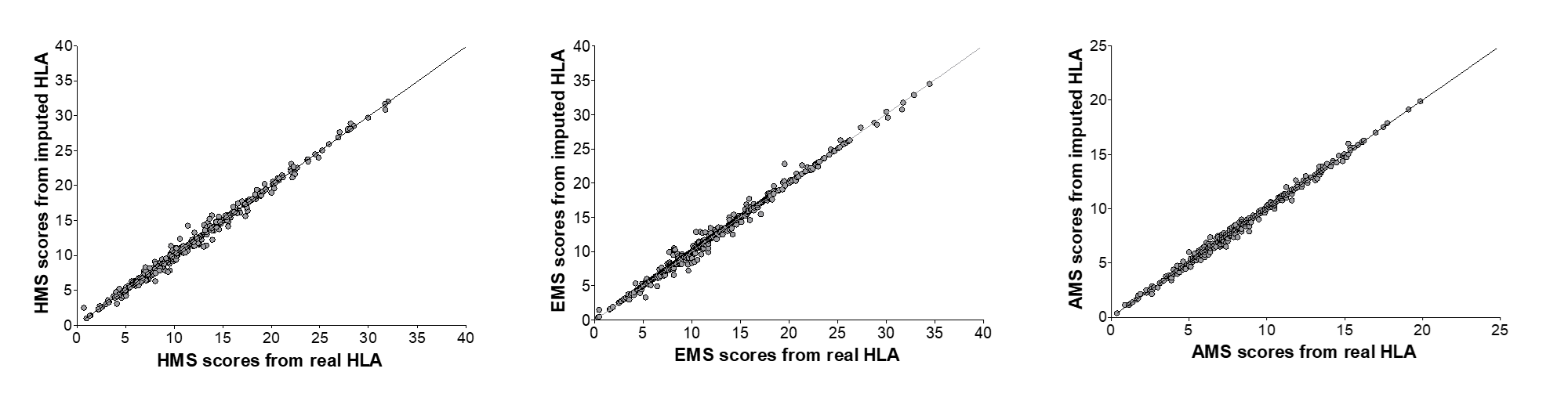
**Supplemental Figure 1.** Comparison of physicochemical immunogenicity scores calculated based on known molecular HLA types and calculated based on HLA types imputed using the HaploStats algorithm.

**Supplemental Figure 2.** Death-censored allograft survival in Blacks and non-Blacks matched by clinical confounders.


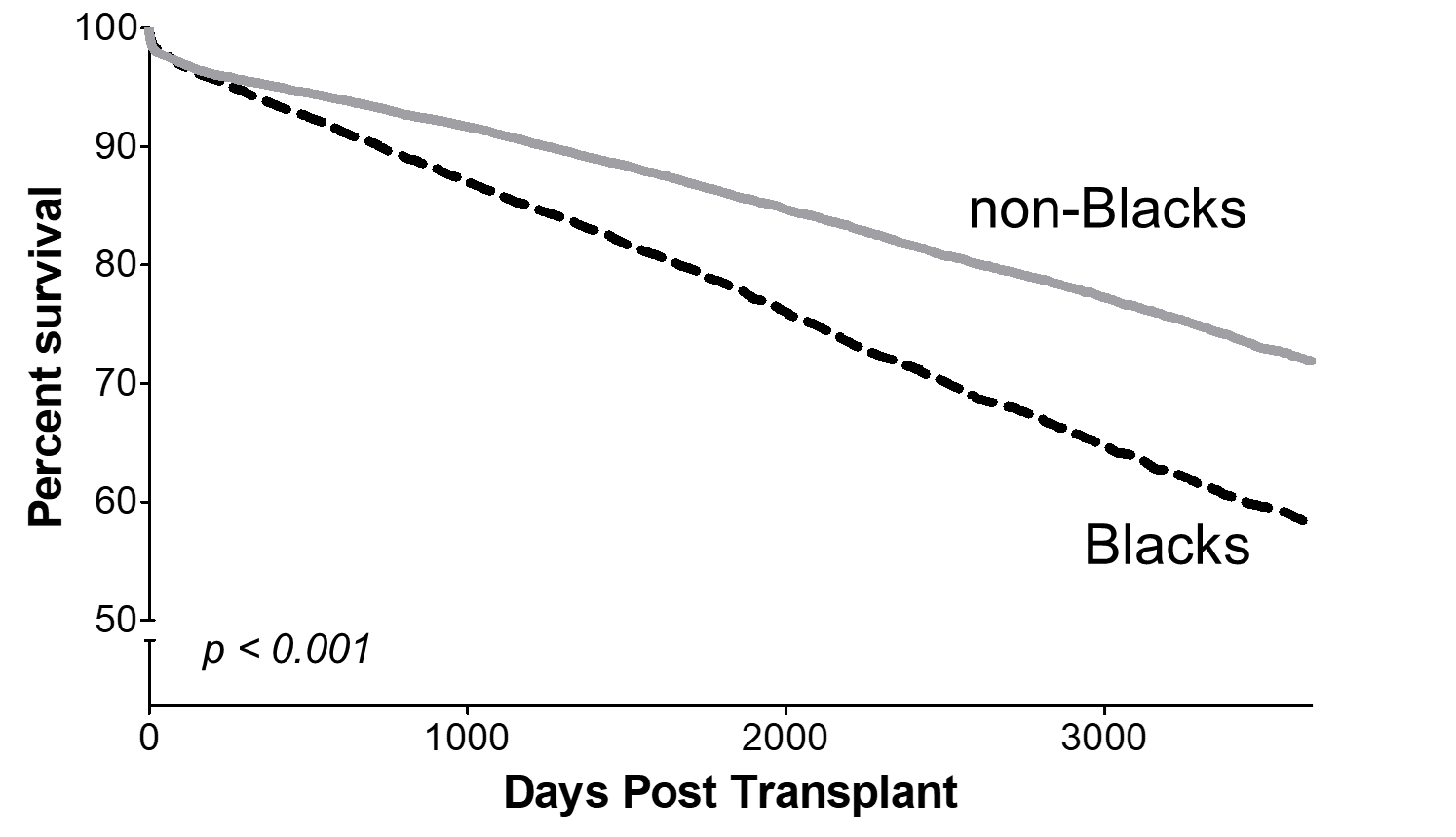


**Supplemental Figure 3.** Death-censored allograft survival in Blacks and non-Blacks stratified by HLA-DR/DQ MM (A, B) in non-Blacks after matching to Blacks (C).


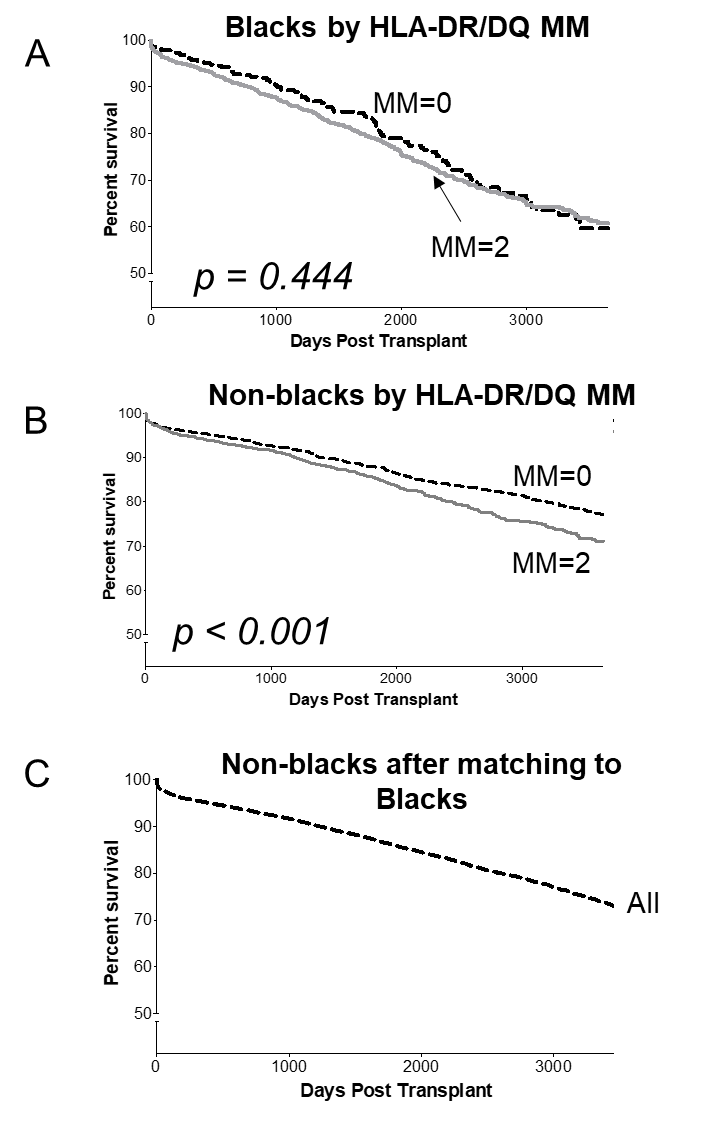


## Supplemental Tables

Supplemental Table 1. Graft survival based on Kaplan-Meier survival estimates (years (95% Confidence Interval)) at immunogenicity categories defined by HLA-A/B/DR HMS score integer cutoffs in Black and non-Black patients.

| **HMS cutoff** | **Stratum** | **Whites** | **Blacks** | **Hispanics** | **Asians** | **Non-blacks** |
| --- | --- | --- | --- | --- | --- | --- |
| 0 | High | 18.4  (18.0… NA) | 12.0  (11.7… 12.6) | 17.1  (15.9… NA) | 17.1  (16.1… NA) | 17.7  (17.1… 18.7) |
|  | Low | 21.6  (NA… NA) | 16.1  (12.3… NA) | 20.9  (NA… NA) | 12.5  (6.2… NA) | 24.0  (NA… NA) |
| 1 | High | 18.4  (17.5… NA) | 12.0  (11.7… 12.5) | 17.1  (15.8… NA) | 17.1  (16.1… NA) | 17.7  (17.1… 18.7) |
|  | Low | 24.1  (NA… NA) | 16.1  (13.9… NA) | 20.3  (NA… NA) | 12.5  (6.2… NA) | 23.1  (NA… NA) |
| 2 | High | 18.3  (17.4… NA) | 12.0  (11.7… 12.5) | 17.1  (15.8… NA) | 17.1  (16.1… NA) | 17.4  (17.1… 18.6) |
|  | Low | 22.7  (NA… NA) | 14.6  (12.3… 16.1) | 21.0  (NA… NA) | 13.0  (12.5… NA) | 22.0  (NA… NA) |
| 3 | High | 18.5  (17.5… NA) | 12.0  (11.7… 12.5) | 17.1  (15.9… NA) | 17.1  (16.1… NA) | 17.4  (16.9… 18.7) |
|  | Low | 22.5  (18.3… NA) | 13.9  (11.9… 15) | 20.4  (NA… NA) | 13.8  (12.5… NA) | 22.0  (18.3… NA) |
| 4 | High | 18.5  (17.4… NA) | 12.0  (11.6… 12.6) | 16.9  (14.5… NA) | 17.1  (16.1… NA) | 17.1  (16.8… 18.6) |
|  | Low | 21.9  (18.0… NA) | 12.9  (11.9… 14.6) | 20.1  (NA… NA) | 13.0  (11.3… NA) | 18.4  (18.3… NA) |
| 5 | High | 18.4  (16.8… NA) | 12.0  (11.6… 12.6) | 16.4  (14.9… NA) | 17.1  (16.5… NA) | 17.2  (16.6… 18.5) |
|  | Low | 21.1  (18.3… NA) | 12.4  (11.8… 13.9) | 18.3  (17.3… NA) | 16.1  (13… NA) | 20.9  (18.3… NA) |
| 6 | High | 18.4  (16.8… NA) | 12.2  (11.7… 12.7) | 16.4  (15.8… NA) | 16.6  (16.5… NA) | 17.1  (16.5… 18.6) |
|  | Low | 21.4  (18.3… NA) | 11.9  (11.4… 12.8) | 17.9  (16.9… NA) | 16.8  (13.8… NA) | 18.4  (18… NA) |
| 7 | High | 18.4  (16.5… NA) | 12.4  (11.7… 13) | 16.4  (14… NA) | 16.6  (16.5… NA) | 17.1  (16.5… NA) |
|  | Low | 18.7  (18.1… NA) | 11.9  (11.5… 12.6) | 17.3  (16.9… NA) | 15.7  (14.3… NA) | 18.7  (17.7… NA) |
| 8 | High | 18.4  (16.2… NA) | 12.3 (11.6… 12.9) | 16.4  (14.4… NA) | 16.6  (16.5… 17.4) | 16.8  (16.5… 18.6) |
|  | Low | 18.2  (18.1… NA) | 12.0  (11.7… 12.7) | 17.3  (16.9… NA) | 16.3  (14.6… NA) | 18.7  (17.7… NA) |
| 9 | High | 16.5  (16.1… NA) | 12.1  (11.3… 12.9) | 16.4  (15.8… NA) | 17.1  (16.6… NA) | 16.6  (16.2… 18.4) |
|  | Low | 18.7  (18.3… NA) | 12.0  (11.7… 12.7) | 17.3  (16… NA) | 16.6  (14.6… NA) | 18.6  (18… NA) |
| 10 | High | 16.5  (15.0… NA) | 12.1  (11.1… 13) | 17.9  (15.8… NA) | 17.1  (14.3… NA) | 16.8  (16.1… NA) |
|  | Low | 18.7  (18.1… NA) | 12.0  (11.8… 12.7) | 17.2  (16… NA) | 16.6  (14.8… NA) | 18.5  (17.5… NA) |

“NA” values indicate confidence interval limits could not be calculated, because they were beyond the follow-up time.

Supplemental Table 2. Multiple Cox regression analysis results.

| **Variable** | **Reference category** | **Category that is compared to the reference** | **Hazard ratio** | **p-value in Cox regression** |
| --- | --- | --- | --- | --- |
| Recipient age at transplant | 18 to 34 years | 34 to 49 years | 0.828 | 0.1384 |
| Peak PRA | 0 to 19% | Over 95% | 1.304 | <0.0001 |
| Dialysis type | No dialysis | Hemodialysis | 1.000 | 0.9976 |
| Donor race | White | Black | 1.548 | <0.0001 |
| Recipient race | White | Black | 1.080 | <0.0001 |
| Recipient BMI>30 | No | Yes | 0.992 | 0.9308 |
| Donor BMI>30 | No | Yes |  |  |
| Donor gender | Female | Male | 0.673 | <0.0001 |
| Recipient primary source of payment | Private insurance | Public insurance | 0.5054 | 0.992 |
| Cold ischemia time>24 hours | No | Yes |  |  |
| Donor age | 18 to 34 years | 34 to 49 years | 1.977 | <0.0001 |
| Recipient gender |  |  |  |  |
| Transplant era | Before 2005 | 2006 and after | 1.061 | <0.0001 |

Supplemental Table 3. Summary of the proportionality verification results. ASSESS statement in SAS Cox regression procedure was used to verify the proportional hazard distribution assumption generating Martingale residuals plots and performing a Kolmogorov-type supremum test on sample plots. Low p-values indicate likely violation of the proportionality assumption.

| **Confounder** | **p-value in Kolmogorov-type supremum test** | **Time-log transformation applied** |
| --- | --- | --- |
| Pre-transplant BMI | <0.0001 | Y |
| Dialysis type | <0.0001 | Y |
| Recipient primary means of payment | <0.0001 | Y |
| Recipient race | <0.0001 | Y |
| Donor age | <0.0001 | Y |
| Donor gender | <0.0001 | Y |
| Donor race | <0.0001 | Y |
| Peak PRA | 0.439 | N |
| Recipient age | <0.0001 | Y |
| Transplant era | <0.0001 | Y |
| HMS | <0.0001 | Y |

Supplemental Table 4. Estimation of gains in kidney life-years for patients matched under every immunogenicity threshold from HMS=0 to HMS≤10.0.

% of transplants refers to the proportion of transplants within the given HMS cutoff.

Current – refers to the % of transplants and graft survival as seen in the retrospective SRTR data.

Simulated – refers to the % of transplants and graft survival seen in the allocation simulations (Section 3.7)

Survival – calculated by multiplying the retrospective survival for this immunogenicity category by the number of transplants.

Gain – calculated by subtracting Current survival from Simulated survival.

|  | **HMS cutoff** | **% of transplants** | | **# of transplants** | | **Survival** | | **Gain** |
| --- | --- | --- | --- | --- | --- | --- | --- | --- |
|  |  | **Current** | **Simulated** | **Current** | **Simulated** | **Current** | **Simulated** |  |
| **Blacks** | 0 | 0.7 | 3.2 | 39.0 | 183.0 | 628.1 | 2,947.4 | 2,319.3 |
|  | 1 | 1.3 | 6.2 | 72.3 | 347.7 | 1,164.2 | 5,601.9 | 4,437.7 |
|  | 2 | 2.6 | 11.0 | 147.3 | 619.1 | 2,149.7 | 9,037.0 | 6,887.3 |
|  | 3 | 5.5 | 18.0 | 310.7 | 1,016.7 | 4,321.5 | 14,141.3 | 9,819.8 |
|  | 4 | 11.1 | 26.0 | 623.6 | 1,467.7 | 8,055.6 | 18,959.5 | 10,903.9 |
|  | 5 | 20.4 | 33.8 | 1,148.6 | 1,903.1 | 14,283.7 | 23,666.2 | 9,382.5 |
|  | 6 | 32.5 | 41.4 | 1,834.8 | 2,333.0 | 21,917.0 | 27,868.7 | 5,951.7 |
|  | 7 | 46.3 | 48.7 | 2,609.9 | 2,745.5 | 31,097.0 | 32,713.1 | 1,616.1 |
|  | 8 | 59.6 | 55.1 | 3,360.2 | 3,104.5 | 40,183.7 | 37,126.9 | -3,056.8 |
|  | 9 | 71.3 | 60.3 | 4,022.5 | 3,400.3 | 48,435.3 | 40,942.9 | -7,492.4 |
|  | 10 | 80.5 | 65.4 | 4,535.8 | 3,689.8 | 54,616.4 | 44,429.2 | -10,187.2 |
| **Whites** | 0 | 8.1 | 17.2 | 555.2 | 1,175.7 | 12,005.9 | 25,423.7 | 13,417.7 |
|  | 1 | 10.5 | 21.2 | 718.7 | 1,449.3 | 17,287.3 | 34,861.4 | 17,574.0 |
|  | 2 | 13.1 | 28.5 | 895.1 | 1,949.8 | 20,280.6 | 44,177.9 | 23,897.4 |
|  | 3 | 18.0 | 36.3 | 1,232.5 | 2,485.2 | 27,705.8 | 55,866.1 | 28,160.3 |
|  | 4 | 26.1 | 44.3 | 1,787.7 | 3,037.0 | 39,133.3 | 66,482.1 | 27,348.8 |
|  | 5 | 37.1 | 51.9 | 2,542.2 | 3,557.6 | 53,561.2 | 74,952.6 | 21,391.4 |
|  | 6 | 49.8 | 58.4 | 3,410.9 | 4,002.2 | 73,124.5 | 85,799.8 | 12,675.3 |
|  | 7 | 62.2 | 64.2 | 4,262.9 | 4,395.1 | 79,733.8 | 82,206.5 | 2,472.7 |
|  | 8 | 73.7 | 68.9 | 5,044.6 | 4,719.2 | 91,577.0 | 85,668.9 | -5,908.1 |
|  | 9 | 82.6 | 72.9 | 5,654.1 | 4,993.5 | 105,755.5 | 93,399.1 | -12,356.4 |
|  | 10 | 88.8 | 76.1 | 6,081.7 | 5,215.0 | 113,753.6 | 97,542.4 | -16,211.2 |
| **Hispanics** | 0 | 2.1 | 2.5 | 69.0 | 85.5 | 1,444.2 | 1,790.5 | 346.4 |
|  | 1 | 3.5 | 3.8 | 116.1 | 127.5 | 2,360.8 | 2,592.2 | 231.4 |
|  | 2 | 5.2 | 6.3 | 173.9 | 211.8 | 3,650.0 | 4,446.1 | 796.1 |
|  | 3 | 8.2 | 10.3 | 276.3 | 347.3 | 5,636.2 | 7,083.8 | 1,447.7 |
|  | 4 | 13.5 | 14.9 | 452.9 | 502.4 | 9,123.9 | 10,120.1 | 996.2 |
|  | 5 | 22.4 | 19.8 | 752.8 | 665.8 | 13,797.8 | 12,202.4 | -1,595.4 |
|  | 6 | 33.8 | 24.7 | 1,137.0 | 830.1 | 20,311.1 | 14,828.2 | -5,482.8 |
|  | 7 | 46.5 | 29.2 | 1,563.0 | 982.6 | 26,968.7 | 16,954.7 | -10,014.0 |
|  | 8 | 58.4 | 34.0 | 1,962.9 | 1,143.8 | 33,870.0 | 19,735.3 | -14,134.6 |
|  | 9 | 69.3 | 38.5 | 2,329.8 | 1,295.0 | 40,199.6 | 22,344.6 | -17,855.0 |
|  | 10 | 78.1 | 42.6 | 2,627.9 | 1,432.1 | 45,143.0 | 24,600.8 | -20,542.3 |
| **Asians** | 0 | 0.9 | 1.1 | 11.4 | 13.7 | 143.0 | 171.6 | 28.6 |
|  | 1 | 1.2 | 2.3 | 15.7 | 29.2 | 196.0 | 364.6 | 168.5 |
|  | 2 | 2.7 | 4.3 | 34.3 | 55.2 | 446.0 | 717.5 | 271.4 |
|  | 3 | 5.1 | 8.0 | 65.3 | 102.3 | 898.8 | 1,407.9 | 509.1 |
|  | 4 | 9.8 | 12.1 | 125.7 | 155.5 | 1,635.5 | 2,022.3 | 386.8 |
|  | 5 | 18.4 | 17.3 | 236.4 | 222.3 | 3,799.7 | 3,573.2 | -226.5 |
|  | 6 | 30.1 | 23.2 | 386.7 | 298.4 | 6,495.7 | 5,012.3 | -1,483.4 |
|  | 7 | 44.0 | 28.8 | 566.3 | 370.9 | 8,914.3 | 5,839.5 | -3,074.8 |
|  | 8 | 57.3 | 33.6 | 737.4 | 432.7 | 11,985.9 | 7,032.9 | -4,953.0 |
|  | 9 | 68.3 | 39.6 | 878.5 | 509.5 | 14,539.0 | 8,433.3 | -6,105.7 |
|  | 10 | 77.5 | 43.6 | 996.0 | 560.7 | 16498.3 | 9287.6 | -7210.8 |
|  | Sum of all gains | 62758.6 | | | | | | |
